# Dietary yeast beta 1,3/1,6 glucan supplemented to adult Labrador Retrievers alters peripheral blood immune cell responses to vaccination challenge without affecting protective immunity

**DOI:** 10.1093/jas/skad029

**Published:** 2023-01-24

**Authors:** Krysten Fries-Craft, Logan R Kilburn-Kappeler, Charles G Aldrich, Elizabeth A Bobeck

**Affiliations:** Department of Animal Science, Iowa State University, Ames, IA 50011, USA; Department of Grain Science and Industry, Kansas State University, Manhattan, KS 66502, USA; Department of Grain Science and Industry, Kansas State University, Manhattan, KS 66502, USA; Department of Animal Science, Iowa State University, Ames, IA 50011, USA

**Keywords:** β-glucans, flow cytometry, Labrador Retrievers, nutritional immunity, peripheral blood mononuclear cells

## Abstract

Yeast-derived 1,3/1,6 β-glucans may alter host immunity to produce robust and quickly resolved responses that align with companion animal health goals. In adult dogs, immunomodulation by yeast 1,3/1,6 β-glucans in extruded kibble diet have not been well documented. The study objective was to evaluate systemic immune responses in dogs fed kibble diets with two yeast 1,3/1,6 β-glucans doses before and after vaccine challenge. Twenty-four adult Labrador Retrievers were assigned to three dietary treatments consisting of a basal diet (control) supplemented with 0.012% or 0.023% (0.5 or 1×, respectively) yeast 1,3/1,6 β-glucan with equal sex representation within each treatment (8 dogs/diet). Animals were fed experimental diets for a 29-d acclimation period, after which baseline blood samples were collected before administration of a combination canine distemper virus, parvovirus, and adenovirus-2 vaccine. Blood samples were collected weekly for 21 d following vaccination with whole blood for CBC analysis, serum for titer and cytokine assays, and peripheral blood mononuclear cells (PBMC) isolated for flow cytometric immune cell profiling. Data were analyzed using the MIXED procedure with diet and timepoint fixed effects. Serum titer was analyzed by Kruskal–Wallis test (SAS 9.4; *P* ≤ 0.05). Prior to vaccination, β-glucan diets did not affect serum cytokines, antibody titer, or immune cell populations. In the first 7 d post-vaccination (dpv), PBMC CD21^low^ B cells increased 36.5% to 58.1% in all groups but the magnitude of change was lesser in the 0.5× β-glucan diet resulting in 25.6% lower CD21^low^ populations compared to control-fed dogs (*P* = 0.007). By 21 dpv, B-cell populations recovered to baseline levels in dogs fed 1× β-glucan, but CD21^high^ cells remained elevated 50.5% in dogs fed 0.5× β-glucan diets compared with baseline (*P* < 0.0001). While no differences in serum titer or cytokines were observed, feeding both β-glucan diets maintained stable blood monocytes, whereas a 53.0% decrease between baseline and 14 dpv was observed in control-fed dogs (*P* = 0.01). Collectively, these outcomes suggest that a 1× dose of 1,3/1,6 yeast β-glucan in extruded kibble diets altered monocytes associated with trained immunity, did not reduce PBMC CD21^low^ B-cell responsiveness, and simultaneously contributed to B-cell population resolution by 21 dpv in adult dogs. Additional research to assess the functionality of these changes is needed.

## Teaser

Yeast 1,3/1,6 β-glucans have shown promise in training human immune responses, but similar outcomes have not been described in adult companion animals. Here, we show potential benefits of yeast 1,3/1,6 β-glucans on canine immunity during vaccine challenge but also identify new areas of emphasis in assessing adult companion animal immunity.

## Introduction

Human and pet food trends are closely intertwined, with trends in one often being reflected in the other (Boya et al., 2015). As such, demand for pet foods with perceived health benefits (e.g., increased immunity) has increased; however, many bioactive ingredients that improve human health are often not assessed separately in companion animals (Schleicher et al., 2019). β-glucans represent a class of potentially bioactive natural polysaccharides from plant, bacteria, and yeast cell walls with varying health outcomes based on biological source and chemical structure. For example, cellulose (1,4 β-glucan) from plants does not exhibit any immunomodulatory effects, whereas such outcomes have been observed from dietary yeast-derived 1,3/1,6 β-glucans (Stier et al., 2014).

Yeast 1,3/1,6 β-glucans are recognized by innate immune cells as microbial-associated molecular patterns and “train” the innate immune system to mount more effective responses without overstimulation (De Marco Castro et al., 2021). Trained innate immune responses are characterized by an increasingly robust response with repeat pathogen exposure but a return to baseline between encounters (Netea et al., 2011; Divangahi et al., 2021). Unlike livestock, companion animals are typically kept for the duration of their natural lifespan and are fed diets corresponding to varying life stages beyond production maintenance. As such, greater importance is placed on promoting immune responses that support long-lasting vaccine protection without contributing to conditions associated with a hypersensitive immune system such as atopic dermatitis (Roth and Spickler, 2010; Bobeck, 2020). In this regard, dietary yeast 1,3/1,6 β-glucans align well with growing consumer trends in pet food and companion animal health goals.

Previous research in dogs has attempted to show such responses in varying contexts. Puppies orally administered fungal 1,3/1,6 β-glucans reached protective antibody titers against canine parvovirus (CPV)-2 and rabies 2–3 weeks earlier than puppies given a placebo (Vojtek et al., 2017). In vitro analysis of β-glucans in canine monocytes supported the presence of trained immunity in this species and subcutaneous injection of algae-derived β-glucans prior to rabies vaccination in beagle puppies increased B cell responses needed to confer protective immunity while also increasing anti-inflammatory interleukin (IL)-10 to potentially mitigate deleterious immune responses (Paris et al., 2020a, 2020b). In adult dogs of varying breeds, dietary yeast β-glucans (0.1%) decreased pro-inflammatory tumor necrosis factor-α but increased pro-inflammatory IL-6 in supplemented obese vs. lean animals (Ferreira et al., 2022). While these studies support the immunomodulatory effects of β-glucans in dogs, they also emphasize discrepancies in source, administration method, immune outcomes, studied life stages (puppy vs. adult) or health statuses (lean vs. obese) that obfuscate-specific immunological outcomes associated with these compounds.

In the current study, proprietary 1,3/1,6 β-glucans extracted from baker’s yeast cell wall were incorporated into extruded kibble diets for adult Labrador Retrievers. Previous research on this specific β-glucan product in adult humans has been linked to reduced upper respiratory infection and allergy symptoms (Talbott and Talbott, 2012; Talbott et al., 2013; Fuller et al., 2017). These outcomes align with companion animal immunomodulatory goals, but incorporation into kibble diet presents challenges in preserving β-glucan presence and bioactivity. Kilburn-Kappeler et al. (2023) showed that this specific yeast 1,3/1,6 β-glucan product could be detected in kibble diets following extrusion processing; however, it is not known if β-glucans inclusion in these kibble diets affects the canine immune system. The study objective was to evaluate immune responses in adult dogs fed yeast 1,3/1,6 β-glucans at two doses (0.5× and 1×) in extruded kibble diets before and after vaccine challenge.

## Materials and Methods

### Animals and diets

All animal protocols were approved by the Institutional Animal Care and Use Committee at Four Rivers Kennel (Nevada, MO; protocol #FRK-34). The immunological outcomes reported herein are part of a dual digestibility and vaccine challenge study (Kilburn-Kappeler et al., 2023). A total of 24 intact, healthy adult Labrador Retrievers (5.1 ± 2.9 years; average body weight 29.1 ± 3.6kg) with equal representation of males and females housed at Four Rivers Kennel were selected for this study. Dogs were randomly assigned to 1 of 3 experimental diets with even distribution of age, weight, and sex within each treatment group (eight dogs/diet). When weather permitted, dogs spent 6–8 h/d in outside yards in a group setting but were housed individually in temperature-controlled kennels overnight and at feeding time (once daily in the morning) with ad libitum access to automated waterers in both locations.

Diets were formulated to be isonutritional, meet AAFCO nutritional requirements for all life stages (ALS), and prepared using extrusion methods reported by Kilburn-Kappeler et al. (2023). Experimental diets consisted of control ± 0.012 or 0.023% 1,3/1,6 β-glucan product corresponding to 0.5× and 1× target doses, respectively (Kerry Group, Tralee, Ireland). The 1× target dose was 1/3 the predetermined dose for adult humans (250 mg/d) from previous research after accounting for body weight (BW) differences and is similar to doses evaluated in children (Talbott and Talbott, 2009; Richter et al., 2015). The half dose (0.5×) was included to evaluate the potential efficacy of lower β-glucan doses in dogs. Post-processing analysis determined 109.5% and 123.2% β-glucan recovery in the 0.5× and 1× diets, respectively, with wheat potentially explaining the surplus β-glucan detected (Kilburn-Kappeler et al., 2023). All diets contained 0.25% fish oil to reflect commercially relevant formulations and meet minimum AAFCO ALS requirements for alpha-linolenic acid, eicosapentaenoic acid, and docosahexaenoic acid. While fish oil as a source of omega-3 polyunsaturated fatty acids may have anti-inflammatory properties, diets in this study had 0.22% omega-3 polyunsaturated fatty acids calculated on a dry matter basis. This low inclusion percentage aligns with control diets used when evaluating therapeutic omega-3 levels for dogs (0.18% vs. 1.47%; Moreau et al., 2012).

Blood was collected from each dog on day 0 to determine serum antibody titer prior to the 24 d feed adaptation period. Daily feed amounts throughout the study were calculated for each dog to maintain BW and exceed nutrient requirements. On day 29 following digestibility study completion (24 d acclimation + 4 d collection), fasted blood samples from the cephalic vein were collected from each dog into serum collection, cell preparation, and EDTA-coated tubes before subcutaneous administration of modified live canine distemper (CDV), CPV, and adenovirus type 2 (CADV-2) combination vaccine (Elanco, Indianapolis, IN). Blood samples from each dog were collected at 7, 14, and 21 d post-vaccination (dpv) with study conclusion on 21 dpv.

### Serum antibody titer and cytokine determination

Approximately 3 mL of blood was collected into BD serum collection tubes (Becton, Dickinson and Company, Franklin Lakes, NJ). Blood rested for 30 min at room temperature in upright tubes before centrifugation at 1,200 × *g* for 10 min. Serum was distributed into two aliquots designated for cytokine and titer analysis. One serum aliquot per dog collected on day 0, day 29 (baseline), 7, 14, and 21 dpv was submitted to the Kansas State University Veterinary Diagnostic Laboratory (Manhattan, KS) for CDV, CPV, and CADV-2 antibody titer analysis and the remaining aliquot was frozen at −80 °C until analysis. Data were reported as the endpoint titer defined as the highest dilution to produce a positive reading.

Cytokine analysis for IL-1β, IL-2, IL-6, IL-10, IL-17A, and interferon (IFN)-γ on day 29 (baseline), 7, 14, and 21 dpv serum samples was completed using commercial canine-specific sandwich ELISA kits (RayBiotech, Peachtree Corners, GA). Standards were prepared according to manufacturer instructions with each kit’s prescribed diluent as the blank (0 ng or pg/mL). Serum samples and standards were plated in duplicate (50 μL/well) on each pre-coated plate. Sample incubation time was increased from the recommended 2.5 to 8 h and biotinylated detection antibody incubation was increased from 1 h to 2 h. All other steps were performed according to the manufacturer’s instructions.

Absorbance data were quantified in Microsoft Excel (version 16.51) using data analysis spreadsheets developed by the kit manufacturer. Briefly, the average blank absorbance was subtracted from all wells and standard curve equations were generated with the intercept set at 0. The resultant standard curve equation was used to calculate the concentration of each cytokine in pg/mL (IL-1β only) or ng/mL. Samples were rerun if cytokines were undetectable in one or both wells.

### Complete blood counts and immune cell profiles

For complete blood count (CBC) analysis, approximately 1 mL of blood was collected into EDTA-coated blood tubes on day 29 (baseline), 7, 14, and 21 dpv and immediately analyzed at Four Rivers Kennel using an Abaxis Vetscan HM5 Hematology Analyzer (Zoetis, Parsippany, NJ). An additional 3 mL blood sample was collected into BD cell preparation tubes with sodium heparin (Becton, Dickinson and Company, Franklin Lakes, NJ). Peripheral blood mononuclear cells (PBMC) were isolated according to manufacturer specifications and frozen in heat-inactivated bovine calf serum with 7.5% DMSO at −80 °C until analysis.

Prior to extracellular staining, cells isolated on day 29 (baseline), 7 and 21 dpv were thawed, enumerated, and aliquoted into 7 flow cytometry tubes/sample. The extracellular flow cytometry panel consisted of unlabeled mouse anti-canine cluster of differentiation (CD) 11b (clone CA16.3E10, IgG1), mouse anti-canine CD21 AlexaFluor 647 (clone CA2.1D6, IgG1), mouse anti-canine CD3-FITC (clone CA17.2A12, IgG1), rat anti-canine CD4 Pacific Blue (clone YKIX302.9, IgG2a), and rat anti-canine CD8AlexaFluor7 00 (clone YCATE55.9, IgG1; BioRad, Hercules, CA) with fluorescence-minus-one and corresponding isotype controls. Rabbit F(abʹʹ)2 anti-mouse IgG-RPE secondary antibody was used to label CD11b antibody. Aliquoted cells were initially stained with unlabeled mouse anti-canine CD11b at manufacturer-recommended dilutions for 30 min at 4 °C in the dark before being washed in PBS and applying secondary antibody under the same incubation conditions. Following secondary antibody application, cells were washed and stained with the remaining markers for 30 min at 4 °C in the dark. Cells were washed and resuspended in PBS before analysis using a BD FACSCanto cytometer. Generated files and cell populations were analyzed using FlowJo 10.5.0 Software.

### Statistical analyses

For all analyses except serum titer, individual dog was used as the experimental unit and data were analyzed using the following statistical model:


$$\begin{array}{l} y_{ijk}=\mu +D_{i}+T_{j}+{\left(D\times T\right)}_{ij}+e_{ijk} \end{array}$$

where *y*_*ijk*_ is the dependent variable, μ is the overall treatment mean, *D*_*i*_ is the effect of diet at the *i*th level (control, 0.5×, 1×), *T*_*j*_ is the effect of timepoint at the *j*th level (day 29 baseline, 7, 14, and 21 dpv), (*D* × *T*)_*ij*_ is the interaction of diet and timepoint, and *e*_*ijk*_ is the random error associated with each observation. Complete blood count, cytokine, and immune cell profile data were analyzed using the MIXED procedure (SAS 9.4, Cary, NC) with Tukey’s post hoc test for least-squares mean separation to account for multiple comparisons. The random effect of dog was used to analyze CBC and cytokine data, while the random effect of tube was used for flow cytometric immune cell data. Outlier immune cell populations were identified using the UNIVARIATE procedure and removed if they were >3 SDs from the treatment mean. No more than 2 data points were removed from each group. Serum endpoint titer is ordinal data on a discrete scale and required analysis using nonparametric tests (Reverberi, 2008). The reciprocal of each dog’s reported endpoint titer dilution was input for analysis by the Kruskal–Wallis test within the NPAR1WAY procedure (SAS 9.4) to determine the diet effect on serum titer with outcomes at each timepoint evaluated separately. For all analyses, significance was denoted at *P* ≤ 0.05.

## Results and Discussion

### Serum antibody titer

**Figure 1.**
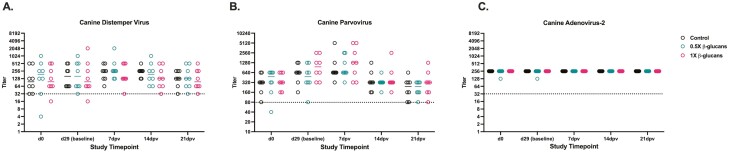
Serum titer in adult Labrador Retrievers fed diets ± yeast 1,3/1,6 β-glucan at 0.5× or 1× doses before study start (day 0), administration of a combination (A) canine distemper, (B) parvovirus, and (C) adenovirus-2 vaccine (day 29), and weekly for 21 d post-vaccination (dpv). Numbers on the x-axis correspond to the reciprocal of the titer ratio (e.g., 32 = 1:32) and each open circle represents individual observations (8 dogs/diet). The dashed line represents the minimum protective titer for each virus. Solid horizontal lines within each diet represent median titer at each timepoint.

Serum antibody titer analysis was conducted prior to study initiation and throughout to assess protective immunity and potential alterations due to dietary yeast β-glucan treatment. No significant differences in serum antibody titer were observed between dietary treatments at any timepoint. The minimum protective titer against CDV and CAV-2 is 1:32 and 1:80 for CPV. On day 0, all dogs met or exceeded minimum titers needed for protection against CAV-2 and CPV, while two dogs assigned to either β-glucan diet had serum titers below the minimum for CDV (Figure 1). After 29 d of feed adaptation, minimal changes in median serum titer were observed and one dog assigned to the 1× β-glucan diet did not have protective titer against CDV (Figure 1A). Peak median titer against CDV and CPV in all groups occurred at 7 dpv, whereas median titer against CAV-2 remained consistent over time (Figure 1). By the study conclusion, median CDV and CPV titer recovered to ratios ≤ day 0 observations in dogs fed either β-glucan diet whereas median CDV titers remained 2.7-fold greater between day 0 and 21 dpv in control-fed dogs (Figure 1A and B).

Biologically relevant change in serum titer against viruses is considered to occur when a 4-fold difference is observed between paired samples (Stowe et al., 2014). Titer changes across treatments throughout this study did not meet this threshold but emphasize some limitations in assessing the immune responses of adult companion animals. Vaccination against CPV, CDV, and CAV-2 is recommended for puppies at 6–8 weeks of age with boosters every 3–4 weeks until 16-weeks-old and subsequent boosters every 3 yr for adult animals (Day et al., 2016; Nova et al., 2018). In a review published by Roth and Spickler (2010), protective titers against CPV, CDV, and CAV-2 can be observed between 3 and 7 yr, indicating that dogs in this study’s age group would have been within the time period of protective immunity at challenge initiation. In addition, CAV-2 is responsible for canine respiratory infections prevalent in group-housed settings (Bergmann et al., 2020). Dogs in this study were allowed to socialize in outdoor group environments and may have been environmentally exposed to CAV-2, contributing to the elevated and unchanged titer observed in this study (Figure 1C). While using an immunologically novel vaccine in this study would have been preferred, options were limited by availability of a serum titer test as opposed to PCR assays designed to detect active infection or serum tests with categorical ‘yes/no’ outcomes.

### Serum cytokines

**Figure 2.**
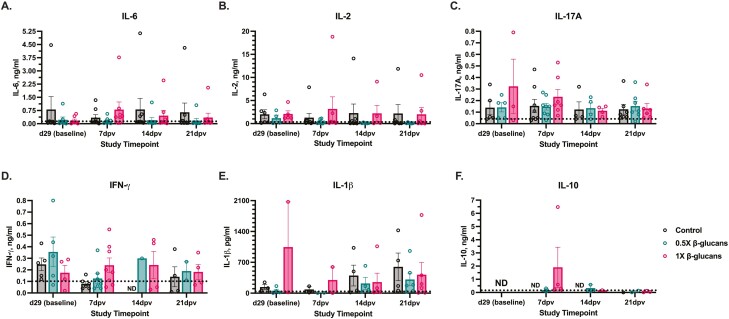
Serum cytokines measured by ELISA in the serum of adult Labrador Retrievers fed diets ± 0.5× or 1× yeast 1,3/1,6 β-glucan before (baseline) and after receiving a combination canine distemper, parvovirus, and adenovirus-2 vaccine. Pro-inflammatory (A) IL-6, (B) IL-2, (C) IL-17A, (D) IFN-γ, and (E) IL-1β were measured along with anti-inflammatory (F) IL-10. Data represent the mean cytokine concentration from 8 dogs/diet ± SEM while plotted points within each group represent individual dogs with detectable cytokine. The dashed line represents the lowest limit of detection for each cytokine determined by the manufacturer as the lowest concentration reliably distinguishable from the assay blank (RayBiotech Inc., Peachtree Corners, GA). ND = not detectable.

Of the pro-inflammatory cytokines measured, IL-1β is associated with innate immune and febrile responses, IL-2 is important for T cell activation and proliferation, IL-6 induces early febrile responses and lymphocyte activation, IL-17A promotes cytokine and antimicrobial peptide secretion, and IFN-γ activates macrophages and increases antigen presentation to the adaptive immune system (Schroder et al., 2004; Iwakura et al., 2008; Dinarello, 2009; Boyman and Sprent, 2012; Tanaka et al., 2014). Anti-inflammatory IL-10 is a potent regulator of the immune system and dampens inflammatory responses by inhibiting macrophage function (Moore et al., 2001). Despite increasing recommended antigen-binding time from 2 to 8 h, serum cytokine detection was highly variable and sometimes below the lower limit of detection (LLOD) specified by the manufacturer as the lowest cytokine concentration that is reliably distinguishable from the blank (Figure 2 dashed lines; Armbruster and Pry, 2008). The two most consistently detectable cytokines in this study were IL-6 and IL-2, while IL-17A and IFN-γ detection varied by timepoint, and IL-1β and IL-10 showed minimal detectability (Figure 2). Due to the high degree of variability between individual animals and sporadic detection, no significant differences in cytokine concentration were observed across dietary treatments. Recently published canine serum cytokine detection often uses multiplex assays capable of detecting multiple cytokines within a sample. While multiplex assays are more sensitive and prevalent, they require specific biomarker validation and specialized equipment, often limiting the cytokines that can be measured through commercially available multiplex kits (Tighe et al., 2015). In contrast, commercially available canine ELISA may not be as sensitive and inter-assay correction values of 15% to 20% are expected, but ELISA kits are available for more cytokine targets. As such, the limited detectability and high variability of cytokine detection in this study may be due to inherent assay limitations with ELISA vs. multiplex assays.

Of the cytokines analyzed in this study, IL-1β and IL-6 are important indicators of trained immunity (Paris et al., 2020b; Divangahi et al., 2021). Prior to vaccination and after 29 d of feed adaptation, IL-6 was detectable in 6–7 dogs/diet while IL-1β was detectable in just 2–4 dogs/diet. After vaccine challenge, IL-6 was detectable in all dogs at 0.1–5.1 ng/mL while detectable IL-1β was observed in ≤6 dogs/diet ranging from 3.2 to 2,072.3 pg/mL (IL-1β LLOD = 10 pg/mL; Figure 2A and E). While IL-1β is not typically included in multiplex panels, serum IL-6 levels in this study were greater than those detected in healthy Labrador Retrievers by multiplex assays when accounting for unit differences (Frank et al., 2015; Richter et al., 2018). This may be due to the detection limits of ELISA vs. multiplex assays, but it is also important to note that a majority of IL-6 throughout this study was at the lower end of the detectable range and often below the LLOD where it would be more similar to published multiplex values. The upper end of the range reported herein is attributed to one dog in the control and 1X diet having detectable IL-6 around 4–6 ng/mL (Figure 2A). This suggests that a more sensitive and reliable assay may be better suited for evaluating the relationship between dietary β-glucans and serum IL-6. While no differences in cytokine concentrations were observed in this study, it is important to note that higher IL-1β and IL-6 in dogs as indicators of β-glucan-associated trained immunity have only been observed ex vivo (Paris et al., 2020a). Future research utilizing more sensitive assays could potentially elucidate the relationship between yeast β-glucans and in vivo cytokine indicators of trained immunity in dogs during vaccination challenges.

### Complete blood counts

In total, 22 parameters were measured by CBC analysis, and all fell within healthy canine reference ranges provided by the hematology analyzer manufacturer (Abaxis, 2018) except for hematocrit and mean corpuscular hemoglobin concentration (MCHC; Table 1). Both measures were similar across dietary treatments, but mean hematocrit was at the upper end of the expected 37.0%–55.0% range and above the upper limit at baseline and 21 dpv. In contrast, MCHC fell below the 31 g/dL reference minimum across all study timepoints (Table 1). Blood collections for CBC analysis in this study occurred in the early morning after dogs were kept in kennels overnight. The mildly elevated hematocrit observed in this study could be a result of dehydration due to overnight fasting (Ashraf and Rea, 2017). Similarly, MCHC is calculated by dividing hemoglobin by observed hematocrit, and the mildly reduced MCHC could be an artifact of increased hematocrit due to dehydration (Gilor and Gilor, 2011).

**Table 1.** Select complete blood count parameters in adult Labrador Retrievers fed diets ± yeast 1,3/1,6 β-glucan at 0.5× or 1× doses

**Parameter**	**Diet**			**Reference range** ^1^	***P*-values**		
	**Control**	**0.5**×	**1**×		**Diet**	**Timepoint**	**Diet × timepoint**
Hematocrit, %							
Day 29 (baseline)	69.0 ± 2.8	66.1 ± 2.8	57.2 ± 2.8	37.0–55.0	0.74	0.004	0.52
7 dpv	53.2 ± 2.8	54.2 ± 2.8	54.4 ± 2.8				
14 dpv	52.7 ± 2.8	54.6 ± 2.8	55.4 ± 2.8				
21 dpv	56.6 ± 2.8	56.9 ± 2.8	56.5 ± 2.8				
MCH, pg							
Day 29 (baseline)	20.6 ± 0.2	20.6 ± 0.2	20.6 ± 0.2	19.5–24.5	0.04	0.23	0.14
7 dpv	20.6 ± 0.2	21.2 ± 0.2	20.6 ± 0.2				
14 dpv	20.8 ± 0.2	21.5 ± 0.2	20.5 ± 0.2				
21 dpv	20.7 ± 0.2	21.2 ± 0.2	20.5 ± 0.2				
MCHC, pg/dL							
Day29 (baseline)	27.3 ± 0.7	28.8 ± 0.7	29.0 ± 0.7	31.0–39.0	0.23	0.39	0.44
7 dpv	28.8 ± 0.7	29.4 ± 0.7	29.0 ± 0.7				
14 dpv	27.3 ± 0.7	29.8 ± 0.7	28.8 ± 0.7				
21 dpv	29.0 ± 0.7	29.2 ± 0.7	28.8 ± 0.7				
Monocytes, %							
Day 29 (baseline)	5.4 ± 0.6^a^	3.8 ± 0.6^ab^	3.4 ± 0.6^ab^	3.0–9.0	0.21	0.22	0.01
7 dpv	3.1 ± 0.6^ab^	2.9 ± 0.6^ab^	5.1 ± 0.6^ab^				
14 dpv	2.5 ± 0.6^b^	3.6 ± 0.6^ab^	3.8 ± 0.6^ab^				
21 dpv	3.7 ± 0.6^ab^	3.6 ± 0.6^ab^	4.7 ± 0.6^ab^				

^1^Reference ranges provided by hematology analyzer manufacturer (Abaxis Inc., 2018).

^2^Reported values represent the mean ± SEM from 8 dogs/diet with equal age and sex distribution.

^a,b^Means with different letter superscripts are significantly different (*P* ≤ 0.05).

Only two of the assessed blood parameters were affected by feeding yeast 1,3/1,6 β-glucans. The diet main effect contributed to higher mean corpuscular hemoglobin (MCH) by 2.2% and 2.7% in dogs fed the 0.5× β-glucan diet compared with control and 1× diets, respectively (*P* = 0.04). In control-fed dogs, monocytes decreased 53.0% from baseline to 14 dpv (*P* = 0.01) but recovered to baseline levels by 21 dpv (Table 1). In dogs, MCH has been shown to increase in response to antibiotics and dietary botanical supplements, but biological implications of this change are often unexplored when values fall within the reference range (Lee et al., 2018; Sainz et al., 2021). Likewise, changes in circulating monocytes as precursors to phagocytic macrophages are typically evaluated in the context of canine disease when values fall outside reference ranges (Bueno et al., 2005). The reduction in monocytes observed only in dogs fed the control diet could indicate that these cells were recruited from the systemic circulation to subcutaneous vaccination sites. In contrast, monocytes maintained relatively stable populations in dogs fed the 0.5× diet or were numerically increased in dogs fed the 1× diet (Table 1). In this context, monocytes are important cells involved in innate immune training and the numerically increased presence observed in this study could be an indicator of trained immunity in adult dogs fed 1× yeast 1,3/1,6 β-glucan (Paris et al., 2020a). However, it is important to note that CBC data provide no functional insights into the “trained” status of these cells and further investigation is needed.

### PBMC immune cell populations

**Figure 3.**
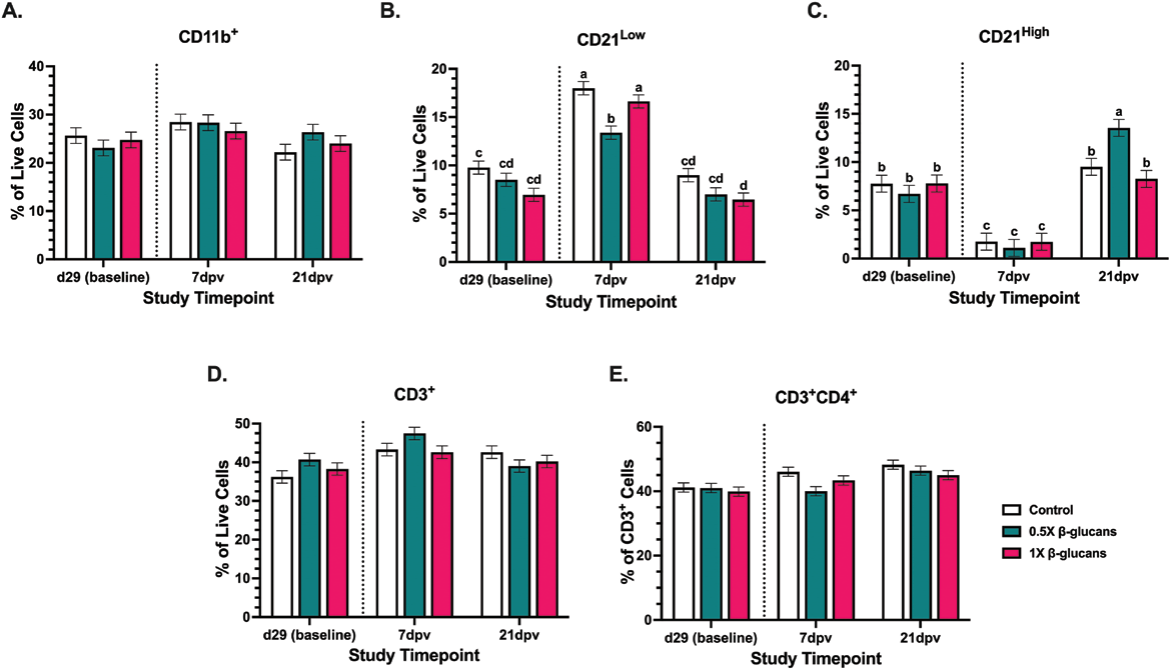
Immune cell populations in the peripheral blood mononuclear cells of adult Labrador Retrievers fed diets ± 0.5× or 1× yeast 1,3/1,6 β-glucan before and after receiving a combination canine distemper, parvovirus, and adenovirus-2 vaccine. Flow cytometry panel was used to analyze innate (A) CD11b^+^ cell, (B&C) CD21^+^ B cell, (D) CD3^+^ T cell, and (E) CD3^+^CD4^+^ helper T cell populations. Populations labeled (B) CD21low and (**C**) CD21^high^ represent CD21^+^ cells with low and high side scatter, respectively. The dashed line separates pre-vaccination baseline immune populations from post-vaccination outcomes. Data represent the mean percentage of cells positive for each marker within live cell or CD3^+^ cell gates (CD3^+^CD4^+^ only) ± SEM. Bars with different letter labels across diets and timepoints are significantly different (*P* ≤ 0.05).

Flow cytometric analysis of PBMC immune cell populations was conducted on 7 and 21 dpv samples to assess early and late vaccine responses. Of the extracellular markers used, CD11b recognizes a mixed population of innate immune cells including granulocytes, monocytes, natural killer cells, and some macrophages (Sherger et al., 2012). Markers for lymphocytes included CD21 on B cells, CD3 on T cells, and CD4 on helper T (T_H_) cells. In this panel, CD21^+^ cells showed distinct grouping at high and low side scatter (SSC) with high SSC being associated with greater structural complexity and a potential indicator of B cell maturity in human studies (Agrawal et al., 2013). Prior to vaccination, canine PBMC were comprised of 24% to 26% CD11b^+^ cells, 6% to 9% CD21^low^ and CD21^high^ B cells, and 36% to 40% CD3^+^ T cells. Immune cell populations within isolated PBMC were unaffected by dietary treatment (Figure 3).

CD11b is the alpha-chain of leukocyte-specific CD11b/CD18 integrin, also known as complement receptor 3. Toll-like receptor stimulation by microbial-associated molecular patterns, such as β-glucans, stimulates CD11b/CD18 activation and consequent intracellular adhesion molecule binding to facilitate immune cell trafficking from peripheral blood into tissues (Gahmberg, 1997). In addition to immune cell trafficking, CD11b/CD18 integrin as complement receptor 3 is vital for the phagocytosis of complement-opsonized pathogens and immune complexes (Dupuy and Caron, 2008). The timepoint main effect increased CD11b^+^ cells 11.9% from baseline to 7 dpv with a 13.0% reduction from 7 to 21 dpv (*P* = 0.001; Figure 3A). While no significant diet × timepoint interactions were observed, the general pattern was for dogs fed the control and 0.5× β-glucan diets to have a numerical 9.9% and 18.5% increase in PBMC CD11b^+^ cells, respectively, from baseline to 7 dpv followed by a numerical 21.9% CD11b^+^ decrease in control-fed dogs from 7 to 21 dpv with maintenance of elevated populations in dogs fed 0.5× β-glucan diets. Such numerical changes within these diets may be underlying the observed timepoint main effect. In contrast, CD11b^+^ PBMC populations were stable across timepoints in dogs fed the 1× β-glucan diet (Figure 3A). This observation suggests that feeding 1× β-glucan potentially reduced reliance on CD11b^+^ cells in early timepoints post-vaccination or cell responses occurred and resolved quickly within the first 7 dpv between sample collections.

In the first 7 dpv, CD21^low^ B-cell populations increased across all dietary treatments (*P* = 0.007). CD21 plays an important role in uptake and retention of immunocomplexes because among other activities, CD21 binds C3 complement fragments and breakdown fragments that remain covalently attached to complement activating surfaces or antigens. The CD21 also promotes activation and survival of memory B cells and aids in development and maintenance of the antibody response to T-cell dependent antigens. In human subject work, CD21^low^ cells are typically characterized as transitional and memory cells in healthy donors and are often increased in conditions with chronic inflammation (autoimmune disease; Thorarinsdottir et al., 2016). CD21^high^ cells have been shown to be more proliferative and produce more antibodies (Suryani et al., 2010). In dogs fed the control and 1× diets, CD21^low^ populations increased 45.7% and 58.1%, respectively, between baseline and 7 dpv, while those fed 0.5× β-glucan showed a lower magnitude 36.5% increase at this time to levels 19.4% to 25.6% lower than the control and 1× diets (*P* = 0.007). This could suggest reduced reliance on CD21^low^ B cells during vaccine responses when dogs were fed 0.5× β-glucan. Despite dogs fed 1× diets having numerically 28.8% lower baseline CD21^low^ populations, CD21^low^ populations in these dogs were similar to the control at 7 dpv, suggesting that CD21^low^ responsiveness following vaccination was unaffected by 1× β-glucan (Figure 3B). In contrast, the timepoint main effect decreased CD21^high^ cells 77.4% to 83.6% from baseline to 7 dpv (*P* < 0.0001; Figure 3C), suggesting recruitment of these cells to the vaccination site. This could indicate quick responsiveness by antibody-producing CD21^high^ B cells to booster vaccination in canines; however, outcomes in this study suggest that β-glucans did not alter CD21^high^ cell responses in the first 7 dpv.

From 7 to 21 dpv, CD21^low^ PBMC populations decreased 47.7% to 61.1% across all dietary treatments (*P* < 0.0001; Figure 3B) to levels similar to each diet’s respective baseline. This suggests uniform resolution of CD21^low^ vaccine responses that were unaffected by dietary β-glucans. At 21 dpv, CD21^high^ populations in dogs fed the control and 1× β-glucan diets were similar to baseline levels whereas dogs fed 0.5× β-glucan had 50.5% increased CD21^high^ cells than those observed at baseline, suggesting a sustained B-cell response (*P* = 0.007; Figure 3C).

Combined, CD11b^+^ and CD21^+^ cell populations in this study suggest general patterns and dose responses to β-glucan supplementation in adult Labrador Retrievers during booster vaccination. Responses in control-fed animals indicate that early immune responses were characterized by a 9.9% numerical increase in CD11b^+^ cells and greater reliance on CD21^low^ memory B cells as indicated by the 45.7% increase compared to baseline in the first 7 dpv (*P* = 0.007; Figure 3A and B). Between 7 and 21 dpv, PBMC CD11b^+^, CD21^low^, and CD21^high^ populations resolved to nearly baseline levels in control-fed dogs. Dogs fed 0.5× β-glucan showed numerically similar CD11b^+^ response patterns to the control and a lower magnitude of CD21^low^ increase in the first 7 dpv but elevated populations of CD21^high^ antibody-producing B cells by study conclusion at 21 dpv (*P* ≤ 0.007; Figure 3A–C). In contrast, dogs fed the 1× β-glucan diet displayed limited reliance on CD11b^+^ cell populations throughout the post-vaccination period with an immune response characterized by a 58.1% increase in CD21^low^ memory B cells in the first 7 dpv to levels similar to the control despite numerically reduced populations at baseline following feed adaptation. By 21 dpv, populations of CD21^low^ and CD21^high^ B cells in dogs fed 1× β-glucan were similar to baseline populations. Altogether, these outcomes suggest that increasing β-glucan dose from 0.5× to 1× in adult dogs reduced immune reliance on CD11b^+^ immune cell populations during booster vaccination without altering CD21^+^ memory B cell responsiveness or contributing to prolonged elevation of CD11b^+^ or CD21^+^ populations in peripheral blood. Combined with titer data, these outcomes indicate that dogs fed 1× β-glucan were able to achieve and maintain protective titer against CDV, CPV, and CADV-2 without altering peripheral immune cell composition beyond 21 dpv.

While B-cell responses were differentially affected by dietary yeast 1,3/1,6 β-glucans, T-cell population shifts were not altered by diet following vaccination (Figure 3D). The timepoint main effect increased peripheral CD3+ T cells 13.6% from baseline to 7 dpv before an 8.6% decrease between 7 and 21 dpv to recover to pre-vaccination levels (*P* < 0.0001). No diet-induced differences in mean population percentages were observed throughout the study (Figure 3D). Minimal T-cell responses in canine peripheral blood in this study were consistent with those observed following booster vaccination in humans (Diks et al., 2021). Underlying these T-cell responses, T_H_ cell subtypes responsible for B-cell activation were evaluated (Masson and Belz, 2010). The timepoint main effect increased T_H_ cells 12.6% between baseline and 21 dpv (*P* < 0.0001; Figure 3E). While no significant diet × timepoint effects were observed, T_H_ cells numerically increased 10.6% in dogs fed the control diet from baseline to 7 dpv while those fed 0.5× β-glucan showed no change until 21 dpv. Combined, these observations indicate that underlying T_H_ cell expansion may contribute to numerical early T-cell expansion in control-fed dogs, but other subtypes may be responsible for a similar outcome in dogs fed diets with 0.5× β-glucan. Initially, the flow cytometry panel in this study included CD8, a marker present on cytotoxic T cells that are important during immune responses to viral pathogens; however, CD8^+^ populations in this study were consistently detected at <1% of CD3^+^ PBMC T cells (data not shown). This low detection was more likely due to problems with anti-canine CD8 binding during the assay rather than altered CD8^+^ T-cell populations. Combined with B cell outcomes, this could also indicate that B-cell responsiveness following vaccination in β-glucan-fed dogs is independent of T_H_ populations.

Outcomes of this study emphasized challenges in researching nutritional immunity in adult canines. In livestock, necropsy to obtain intestinal tissues can be done to ascertain tissue-specific immune responses to dietary components. This sampling strategy is not used in companion animals due to ethical concerns and collecting intestinal biopsies during an endoscopy to gain similar insight is invasive and involves anesthesia-associated risks. As a result, immunological assessment is limited to systemic compartments in peripheral blood; however, lack of systemic response does not discount the possibility of meaningful tissue-level immune responses. Likewise, growth changes can be used in livestock to determine severity of immune challenges and the subsequent protective effects of feed ingredients during challenge but cannot be applied similarly to adult companion animals as this life stage is associated with body weight maintenance. Finally, vaccine challenges using attenuated viruses and bacteria offer a relatively safe way to assess immune responses but are best implemented in young, immunologically naïve animals. Previous research examining nutritional immunomodulation during vaccine challenge in companion animals is primarily done in puppies around 2-3 mo old (Kandil and Abou-Zeina, 2005; Vojtek et al., 2017). As such, potential vaccines available for similar application in adult companion animals are limited to those that are not part of routine puppy vaccination schedules, do not confer long-lasting immunity (<1 yr), and have available quantitative titer tests. These limitations in assessing canine nutritional immunity are of particular concern as companion animals are typically kept for the duration of their natural lifespan and a majority of this time is spent in adult life stages (Wallis et al., 2018). However, use of an adult canine model and a routine vaccine in this study closely represents real-life situations that occur frequently as part of companion animal ownership. As such, the limitations emphasized herein are not intended to discourage investigation of companion animal nutritional immunity or application of a similar adult canine/routine vaccination challenge model. Instead, future work to improve this model should emphasize the development of additional quantitative serum titer analyses for routinely administered canine vaccines and increase or improve existing reagents (e.g., ELISA kits, multiplex biomarker validation, flow cytometry antibodies) to broaden insights that can be gained from peripheral blood.

Despite these limitations, cell-based analyses indicated that feeding higher 1,3/1,6 β-glucan (1× vs. 0.5×) numerically increased monocytes as a potential indicator of trained immunity; however, IL-1β and IL-6 as cytokine indicators of this type of immune response were highly variable between dogs and difficult to assess. Outcomes in CD11b^+^ cells, which include monocytes, indicated that this cell type either responded too quickly to detect any change at 7 dpv in dogs fed 1× β-glucan or was not a critical component of the immune response to booster vaccination in these dogs. Prior to vaccine challenge, 1× yeast 1,3/1,6 β-glucan numerically reduced populations of CD21^low^ B cells compared with the control but did not affect the responsiveness of this cell type to vaccination challenge and did not contribute to prolonged CD21^+^ B-cell population responses as observed in 0.5× β-glucan diets. These outcomes support the use of 1 × 1,3/1,6 β-glucan in extruded kibble diets to improve immune responsiveness without contributing to sustained and potentially detrimental inflammation, but further investigation is recommended.

## Abbreviations

ALS: all life stages
BW: body weight
CADV: canine adenovirus type 2
CBC: complete blood count
CD: cluster of differentiation
CDV: canine distemper virus
CPV: canine parvovirus
DPV: days post-vaccination
IFN: interferon
IL: interleukin
LLOD: lower limit of detection
MCH: mean corpuscular hemoglobin
MCHC: mean corpuscular hemoglobin concentration
PBMC: peripheral blood mononuclear cells
SSC: side scatter
T_H_: helper T cell
